# Non-Destructive Examination for Cavitation Resistance of Talc-Based Refractories with Different Zeolite Types Intended for Protective Coatings

**DOI:** 10.3390/ma16165577

**Published:** 2023-08-11

**Authors:** Milica Vlahović, Ana Alil, Aleksandar Devečerski, Dragana Živojinović, Tatjana Volkov-Husović

**Affiliations:** 1 Institute of Chemistry, Technology and Metallurgy, National Institute of the Republic of Serbia, University of Belgrade, 12 Njegoševa St., 11000 Belgrade, Serbia; ana.alil@ihtm.bg.ac.rs; 2 Vinča Institute of Nuclear Sciences, National Institute of the Republic of Serbia, University of Belgrade, 12-14 Mike Petrovića Alasa St., Vinča, 11351 Belgrade, Serbia; drak@vin.bg.ac.rs; 3 Faculty of Technology and Metallurgy, University of Belgrade, 4 Karnegijeva St., 11000 Belgrade, Serbia; gaga@tmf.bg.ac.rs

**Keywords:** cavitation erosion, image analysis, principal component analysis, degradation level, morphology analysis

## Abstract

In many industrial processes that include fluid flow, cavitation erosion of different engineering structures (pumps, turbines, water levels, valves, etc.) during their operation is expected. Metallic, ceramic, and composite materials are usual candidates considered for application in such extreme conditions. In this study, the idea is to synthesize refractory ceramic material based on talc with the addition of zeolite for utilization as protective coatings in cavitating conditions. Two talc-based refractories with zeolites from two Serbian deposits were produced. The behaviors of the samples in simulated cavitation conditions were examined by an advanced non-destructive methodology consisting of monitoring mass loss and surface degradation using image analysis compiled with principal component analysis (PCA), interior degradation by ultrasonic measurements, and the microstructure by a scanning electron microscope (SEM). Lower mass loss, surface degradation level, and modeled strength decrease indicated better cavitation resistance of the sample with Igros zeolite, whereby measured strength values validated the model. For the chosen critical strength, the critical cavitation period as well as critical morphological descriptors, Area and Diameter (max and min), were determined. A Young’s elasticity modulus decrease indicated that surface damage influence progressed towards interior of the material. It can be concluded that the proposed methodology approach is efficient and reliable in predicting the materials’ service life in extreme conditions.

## 1. Introduction

Engineering materials are used for constructing various industrial equipment or parts of different shapes and sizes. The most common engineering materials are steel and ceramics, especially refractories. Depending on the planned applications, the necessary selection of materials relies on numerous factors such as mechanical and chemical properties, dimensional tolerance, component shape, manufacturing and service requirements, and cost [1,2]. The basic principles for the selection of material are behavior in different operating conditions and its life cycle. Very often during service life, equipment or some of its parts are exposed to extreme conditions. In this respect, apart from investigating cavitation’s influence on the performance of ceramic matrix composites, metal matrix composites, and alloys [3,4,5], some authors’ previous studies were also focused on changes in composite materials induced by aggressive acid and salt environments [6], high temperatures [7], thermal shocks [8], as well as by laser beams [9]. Failure analysis is usually performed on different construction materials, such as metals, alloys, and refractories [10,11].

Fluid flow systems are often places where cavitation erosion is expected. A cavitation phenomenon involves the formation of water vapor bubbles, which cause (metal/ceramic/polymer/composite) component damage when they collapse back into the liquid phase [12,13,14,15,16]. The sources of heat that contribute to excessively high temperatures and cavitation in a system’s fluid may include high working temperature, turbulent flow conditions in conduits, heat of vaporization in cavitation flow, high-pressure drops across control orifices, high friction from rough surfaces and abrasive action, etc. Cavitation phenomena in metallurgical furnaces and reactors could be associated with the high temperature, turbulent fluid flow, high friction from different surfaces, and vaporization [17,18,19,20]. The level of erosion caused by cavitation could be severe and could affect the fracture initiation, progression, and failure of the part. This phenomenon is extremely important for engineering materials and structures, as it has a strong impact on the lifetime of materials used and thus on the reliability of equipment, structures, or even plants. Accordingly, many investigations have been related to cavitation erosion understanding, and those up to date are focused on new and advanced materials for different applications. A variety of materials, such as metals [12,13,14,15,17,18,19], ceramics [20,21,22,23,24], polymers [25,26], nanomaterials [16], and composites [27,28,29], were investigated for their possible applications in cavitation conditions.

Some of the conditions related to cavitation are present in metallurgy, especially in different processes that could be characterized by high temperatures and high levels of erosion caused by solid (solid particles in furnaces, or flue gas), liquid (liquid metals or alloys), and gas (flue gas), influencing the refractory lining. Refractory lining in most furnaces is built by using various ceramic materials for the different zones depending on the process, temperature, and chemical regime.

There are many conventional and modern methods for ceramics production. Methods that are mainly utilized nowadays are cold pressing (CP), hot pressing (HP), hot isostatic pressing (HIP), spark plasma sintering (SPS), flash sintering (FS), and microwave (MW).

Cold pressing is a simple and economical technique that is still used in mass production industries. This process includes using compaction pressure in a mold (usually made of steel) at room temperature. The pressing is followed by pressure-less sintering so that the pressed ceramic part reaches the final state.

Hot pressing is based on using a uniaxial pressure (10–30 MPa) through a mold at the sintering temperature, which accelerates the densification, thus improving the overall material’s density and even getting close to the theoretical value in a reasonable time as well as providing a significant grain size reduction [30,31]. Hot isostatic pressing implies concurrent application of high temperature (up to 2200 °C) and a gas pressure (200–500 MPa) identically in all directions, usually through an impermeable membrane to compact the ceramic component. It provides producing fully dense parts and achieving practically theoretical density with limited grain growth. For example, post-sintering treatment by HIP is applied to eliminate residual porosity during production of transparent ceramic parts. This technique is mainly used for engineered ceramics requiring optimum properties for high-performance applications, such as glass–ceramic nuclear wasteforms [30,32,33].

Spark plasma sintering is an advanced processing technology that has been developing significantly in the last three decades. This is a synthesis and processing method that enables sintering and sinter bonding at low temperatures and in short periods by discharging between the powder particles’ surfaces and/or secondary in gas discharge and Joule heating [30]. Enhanced densification is attributed to the elimination of the adsorbed gas and impurity because of the spark’s discharge.

Advanced ceramic materials such as nano-structural ceramics, functionally graded materials, fine ceramics, ceramic matrix composite materials, new wear-resistant materials, thermoelectric semiconductors, and biomaterials are produced by this technique. Due to the accelerated densification and the fast-sintering cycles, SPS has become a leading method for producing transparent ceramic materials. The advantage of this method over others is that homogenous highly dense sintered compacts with finer microstructures are obtained faster and at lower temperatures than by conventional sintering methods [34,35,36,37].

Flash sintering is a novel technique for ceramic processing in a conventional sintering furnace at low temperatures in a very short time by the application of an electric field. The specimen is attached to two platinum electrodes, allowing the passage of current. A specimen temperature enhance encourages the conductivity raise, enabling sintering within seconds. High fields allow sintering to high densities almost instantly above a critical temperature.

The significant reduction in temperature and time makes this technique a more energy-efficient alternative for materials processing and relevant in case of the presence of volatile elements in the material [38,39].

The microwave technique involves high heating rates and rapid processing times with direct homogeneous heating of the material by electromagnetic radiation through energy conversion. This nonconventional technique has been successfully developed to fulfill industry demands for reducing energy consumption and processing time, as well as for improving materials properties [40,41].

Considering technical and economic parameters, refractory coatings are often used for different zones in furnaces, and the most-utilized protective coating is related to foundry production [42]. As the coating is in direct contact with the hot metal stream, cavitation testing can provide useful information regarding the material’s behavior and lifetime under these conditions. A lifetime of refractories, including protective coatings, is a very important techno-economical parameter. Prolongation of the lifetime of refractory lining could be provided using different coatings. In this paper, the attempt was to use classical cavitation testing in water to measure the level of degradation. Similar conditions with significant degradation level for refractory service could be expected with gases and liquid metal flows. The obtained results could be implemented for prediction of the degradation level of a refractory coating in contact with a hot metal stream and correlated with other mechanical properties, such as mechanical strength.

For the assessment of refractories’ behaviors during their service lives, various destructive and non-destructive inspection methods, including image and principal component analyses, as well as ultrasonic measurements, can be applied in simulated real conditions, in this case, under cavitation exposure.

A variety of testing techniques found in many disciplines produce large datasets that should be analyzed to obtain results. To offer the appropriate interpretation of such datasets, many different methods have been developed with the aim of reducing their dimensionality. One of the oldest and most widely used techniques is a multivariate statistical analysis- principal component analysis (PCA). Its idea is simple; reduce the dimensionality of a dataset while preserving as much “variability” (i.e., statistical information) as possible. PCA is used for standardization of the range of continuous initial variables, computation of the covariance matrix to identify correlations, computation of the eigenvectors and eigenvalues of the covariance matrix to identify the principal components, creation of a feature vector to decide which principal components to keep, and recast the data along the principal components’ axes [43,44,45].

**Figure 1:**
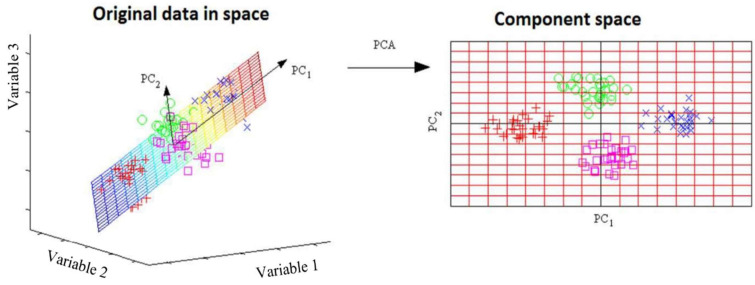
Transformation of original variables from three-dimensional to two-dimensional space, adopted from [45].

The first task of the PCA is to determine the linear combination of the original variables that will have the maximum variance. Another task is to determine several linear combinations of the original variables, which, in addition to having the maximum variance, will be mutually uncorrelated, losing as little as possible of the information contained in the set of original variables. In the process of applying this method, the original variables are transformed into new variables (linear combinations) called principal components. This transformation is obtained by rotating the variables, as shown in Figure 1. Objects are shown with circles, crosses, squares, and pluses.

In Figure 1, the principal components are identified in the plane that optimally describe the largest variance of the data. This space after rotation can be represented as a two-dimensional component space. Coefficients close to zero suggest that the corresponding original variable does not significantly participate in the formation of the principal component.

As the refractories based on talc have low hardness, an attempt to strengthen the material by adding zeolite and thus improve their cavitation resistance was made in this study. The aim was to investigate and compare behavior of synthesized and afterward sintered refractory samples with zeolites from two deposits in simulated cavitation conditions using the advanced methodology for characterizing the material performance without causing damage. This methodology is based on non-destructive evaluation that includes image analysis with principal component analysis and ultrasonic measurements, combined with scanning electron microscopy. By compiling different approaches, the possibility of using talc-based refractories with zeolite as protective coatings under cavitation conditions will be assessed.

## 2. Experimental Methods

### 2.1. Materials

#### 2.1.1. Talc

The talc used in this work was excavated at the Studenica site in Serbia.

Talc was characterized by high inertness and resistance to acids, alkalis, and heating. This mineral is extremely soft, precisely the softest with a hardness of 1 on the Mohs scale, and thus easy to grind [46].

The preparation consisted of combined procedures of mineral processing (leaching and comminution) of raw talc. Special attention was paid to the purification of talc by leaching and lowering the content of Fe_2_O_3_ and CaO. Afterward, the obtained product was subjected to a grinding process in a vibrating mill to an average grain size of 25 μm.

The chemical composition and properties of the starting talc powder used in the experiment are shown in Table 1 and Table 2, respectively.

#### 2.1.2. Zeolite from the Igros Deposit

The results of the complete chemical analysis of the zeolitized tuff from the Igros deposit (Serbia) are given in Table 3.

#### 2.1.3. Zeolite from the Zlatokop Deposit

The average chemical composition of zeolitic tuffs from the Zlatokop deposit (Serbia) is shown in Table 4.

#### 2.1.4. Samples Preparation

In this study, two types of refractory samples were prepared based on talc (85%) and zeolite (15%) from two deposits, Igros and Zlatokop. Sample fabrication consisted of mixing, pressing, and sintering talc and zeolite powder. All components were milled to a particle size of up to 25 μm.

Talc and zeolite powder were mixed in the given ratio and then pressed to form cylinder-shaped samples. The samples were sintered at a temperature of 1200 °C. The sintering process took place according to the following regime: heating to a temperature of 1000 °C at a heating rate of 5 °C/min (during 200 min); heating from 1000–1200 °C, with a heating rate of 2 °C/min (during 100 min); sintering of the sample at a temperature of 1200 °C for 60 min; and cooling the sample in the furnace. The radius and height of the cylindrical samples were 15 mm and 5 mm, respectively.

#### 2.1.5. Characterization of Manufactured Refractory Samples

The phase purity and crystallinity of obtained samples were examined using X-ray diffraction (Rigaku Ultima IV, Tokyo, Japan). The X-ray beam was nickel-filtered CuKα1 radiation (λ = 0.1540 nm, operating at 40 kV and 40 mA). XRD data were collected from 5 to 80° (2θ) at a scanning rate of 5°/min. Phase analysis accompanied by Rietveld refinement was performed using the PDXL2 package software (version 2.8.4.0), with reference to the patterns of the International Centre for Diffraction Database (ICDD PDF-2 2023).

**Figure 2:**
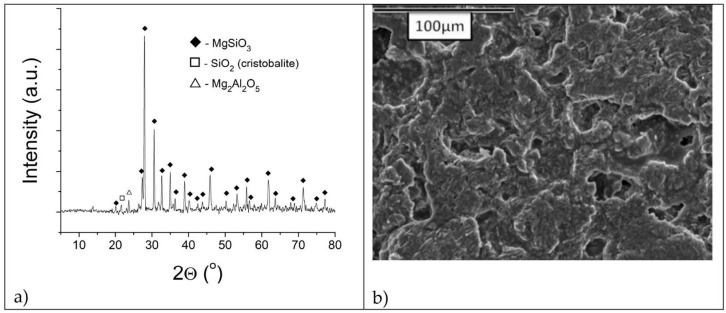
Talc-based refractory sample with zeolite Igros sintered at 1200 °C: (**a**) diffractogram; (**b**) SEM microphotograph.

**Figure 3:**
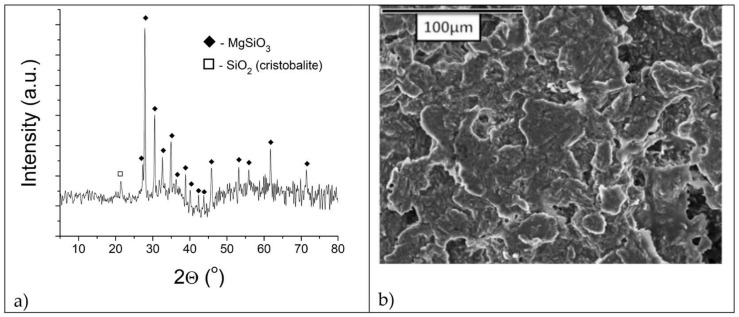
Talc-based refractory sample with zeolite Zlatokop sintered at 1200 °C: (**a**) diffractogram; (**b**) SEM microphotograph.

Figure 2a,b show XRD and SEM analyses of a refractory talc-based sample with zeolite Igros, respectively, whereas XRD and SEM analyses of a refractory talc-based sample with zeolite Zlatokop are presented in Figure 3a,b, respectively.

According to Figure 2a and Figure 3a, the XRD patterns of both samples are almost identical, showing a MgSiO_3_ phase (DB card number: 01-075-1720) with some traces of SiO_2_ (DB card number: 01-076-0934) and Mg_2_Al_2_O_5_ (DB card number: 00-061-0324).

XRD quantitative analysis results, obtained by Rietveld method, are given in Table 5.

The absence of the stronger peaks of Al-rich crystalline phases in both samples indicated that Al was incorporated into the MgSiO_3_ lattice.

SEM microphotograph presented in Figure 2b shows the fine structure of the refractory sample with Igros zeolite, whereas the SEM microphotograph of the refractory sample with the Zlatokop zeolite indicates its homogeneous structure without the presence of porosity (Figure 3b).

### 2.2. Methods

#### 2.2.1. Cavitation Erosion Testing

Prepared refractory samples were subjected to the cavitation erosion test using an ultrasonic vibratory method (with stationary specimen) according to the ASTM G-32 standard [47]. Cavitation testing was performed in intervals of 10 min with a total testing time of 80 min. The selection of characteristic parameters for this method such as waveguide vibration frequency, liquid temperature, the distance of the sample from the front surface of the probe, and liquid properties were chosen in accordance with the standard.

**Figure 4:**
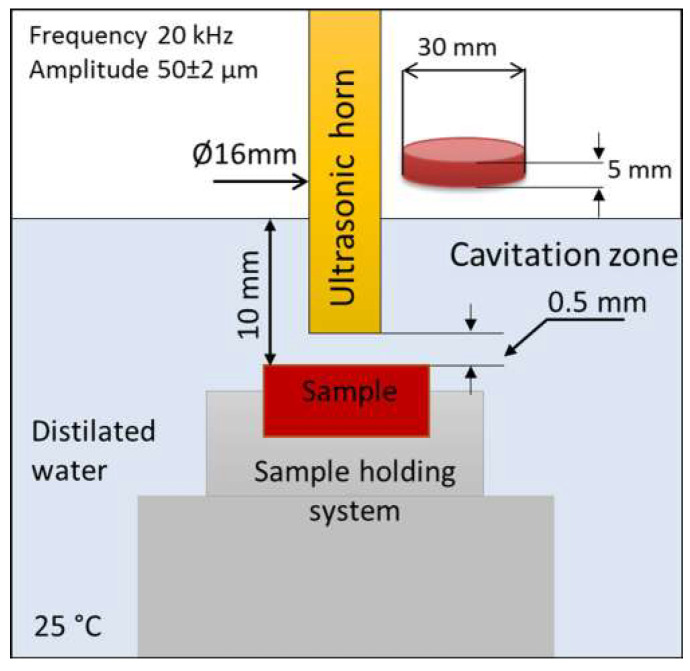
Schematic detail of cavitation vibratory setup.

The cavitation setup with relevant characteristics is given in Figure 4.

Before and after each interval of the cavitation exposure, the refractory samples were dried in the oven at 110 °C to constant mass and then subjected to characterization. All the obtained results are average values of three replicate measurements.

#### 2.2.2. Monitoring the Effects of Exposure to Cavitation

##### Macroscopic and Microscopic Measurements

Before the cavitation test, as well as after each testing period, the samples were photographed using a scanner with the appropriate resolution (1200 dpi) (OM). Photographs were subjected to image analysis using Image-Pro Plus 6.0 software package (Media Cybernetics, 2006, Rockville, MD, USA) to monitor the surface destruction level as well as to perform morphological analysis of the formed pits during the cavitation process.

The microstructural changes in the samples during the cavitation test were monitored using scanning electron microscope type “JEOL” model JSM 6610 LV. The preparation of the samples consisted of coating them with gold.

##### Mass Measurement

Determination of mass loss is the initial step of the usual procedure in examining the cavitation impact on the material and represents the first indicator of the erosion level.

Masses of the samples before and after each interval of the cavitation testing were measured using an analytical balance with an accuracy of ±0.1 mg. The measurement was performed individually for each sample after every 10 min of testing the cavitation effect, for a total testing time of 80 min.

##### Image Analysis

The degree of cavitation-induced damage at the sample’s surface was monitored nondestructively, by image analysis employing Image-Pro Plus software.

Digital photos obtained by OM (scanner) were analyzed, and, by periodical measuring of the damaged surface area during the testing, the destruction level was determined [8,48].

Also, Image-Pro Plus software offered the determination of many descriptors that provide a morphological characterization of formed pits [49]. The list of morphological descriptors selected in this study is given in Table 6.

##### Principal Component Analysis

Processing many parameters (descriptors) by image analysis software is not the best solution for some cases. A multivariate statistical technique, principal component analysis (PCA), and approach could provide fewer relevant parameters, making analysis faster and more reliable.

Principal component analysis was performed for additional analysis and selection of morphological descriptors, to determine their influence on the samples’ behavior. PCA was used with the goal to achieve a better correlation between the morphological descriptors and their influence on formed pits because of the sample degradation due to cavitation erosion. PCA was performed for the selected morphological descriptors given in Table 6. The original dataset of the measured values of descriptors for isolated pits obtained using Image-Pro Plus software was subjected to PCA to establish a correlation between the parameters for examined periods.

##### Ultrasonic Measurements

**Figure 5:**
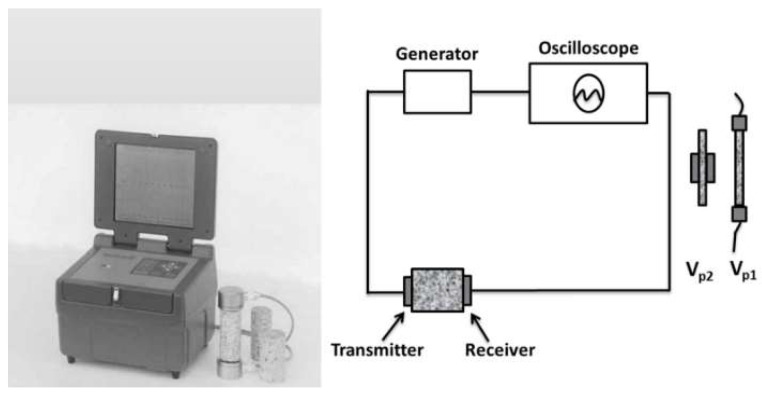
Set up for ultrasonic measurements.

The damage inside the refractory samples, caused by the cavitation, was indirectly determined based on an ultrasonic non-destructive method that measured wave propagation velocities through the material according to the standard procedure SRPS B.B8.121 and using the device model OYO 5210 (Figure 5) [8].

The ultrasonic method involved the use of special devices that emitted ultrasonic waves and registered the time for these pulses to travel a known distance through a material. This method was based on the principle that the ultrasonic wave (pulse) travels at a lower speed through media of higher porosity, i.e., of lower density. Lower values of pulse velocities led to lower values of Young’s elasticity modulus, thus indicating weaker elastic properties of such a material. In the majority of cases, these devices are designed on the principle of transmission of longitudinal ultrasonic waves since it ensured the highest measurement accuracy [50].

The occurrences of micro and macro crackles had an impact on the velocity of wave propagation through the material, as well as on the value of its Young’s elasticity modulus. The development of damage could thus be monitored directly by measuring some of the mentioned mechanical characteristics. Changes in the material structure could be monitored in detail by passing the ultrasonic pulse generated by the transmitter through the sample placed in the test cell, i.e., by measuring the pulse velocity.

Pulse velocity (V) is calculated from the expression (Equation (1)) [51]:

V = L/T (1)

where

L is the path length (m), and T is the time taken by the pulse to traverse that length (s).

The velocity of longitudinal ultrasonic waves (*V_p_*) through the material is a function of some of its physico-mechanical properties, namely, dynamic Young’s modulus of elasticity, dynamic Poisson’s ratio, and density.

These physical constants of the material are in a certain mutual dependence but also in a functional relationship with its structural properties such as porosity and strength. This functional dependence is predominantly expressed on the relation between density and physico-mechanical properties, thus the ultrasonic method can be applied to determine all properties related to density.

The calculation of dynamic Young’s modulus of elasticity (*E_dyn_*) is also enabled using ultrasonic pulse velocities as follows (Equation (2)) [50,51]:

(2) $$E_{dyn}=V_{p}^{2}\;\gamma \;\frac{\left(1+\mu_{dyn}\right)\left(1-2\mu_{dyn}\right)}{1-\mu_{dyn}}\;\;\;\;(\mathrm{Pa})$$

where

*V_p_* is longitudinal pulse velocity (m/s); *γ* is density (kg/m^3^); and *μ_dyn_* is dynamic Poisson’s ratio.

Lower values of pulse velocities lead to lower values of Young’s elasticity modulus, thus indicating weaker elastic properties of such a material.

## 3. Results and Discussion

### 3.1. Macrostructure and Microstructure Analysis

**Figure 6:**
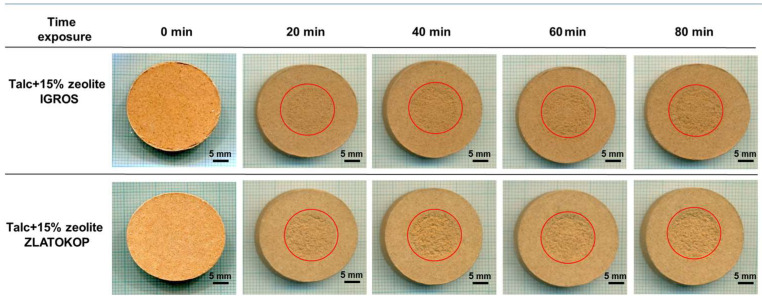
Macrophotographs of talc-based refractory samples with Igroš zeolite and Zlatokop zeolites, before and during cavitation exposure.

Macrophotographs of the refractory talc-based samples with zeolite before and during the cavitation testing are displayed in Figure 6.

According to Figure 6, damage propagation with increasing cavitation exposure time of both talc-based refractory samples is evident, whereby those with Zlatokop zeolite were more destructed.

**Figure 7:**
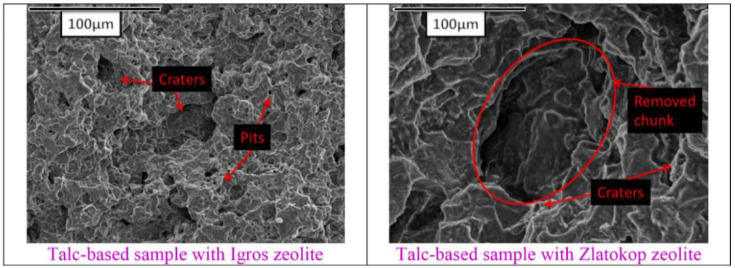
SEM images of the refractory samples after 80 min of cavitation testing.

SEM images for the refractory samples after 80 min of testing are given in Figure 7.

As seen in Figure 7, unlike the sample with zeolite Igros, the sample with zeolite Zlatokop after the same period of exposure to cavitation (80 min) showed severe erosive damage with craters and chunks of removed material.

Detailed information about the pits’ formation and the morphology of the resulting pits were obtained using morphological analysis.

### 3.2. Mass Loss

**Figure 8:**
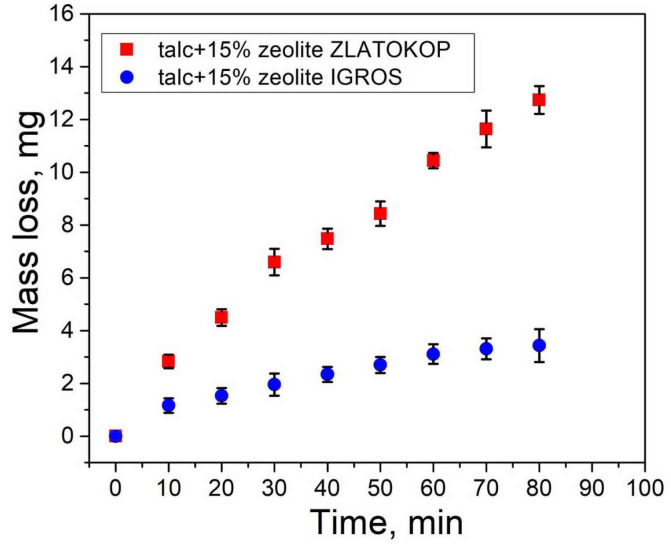
Mass loss of talc-based refractory samples with zeolites.

The mass loss of talc-based refractory samples with 15% zeolite Igros and 15% zeolite Zlatokop is shown in Figure 8.

As seen in Figure 8 the mass loss of both refractory samples increases with the increasing testing time. Larger values for mass loss were measured for a sample with zeolite “Zlatokop”. A slight rise in the mass loss for a sample with zeolite “Igroš” during the testing indicated its better resistance to cavitation. The dependence mass-loss time was continual, and, therefore, cavitation periods could not be accurately distinguished.

### 3.3. Monitoring the Surface Damage by Image Analysis

The refractory samples before and during the cavitation exposure were photographed and subjected to image analysis using Image-Pro Plus software.

#### 3.3.1. Surface Degradation Level

**Figure 9:**
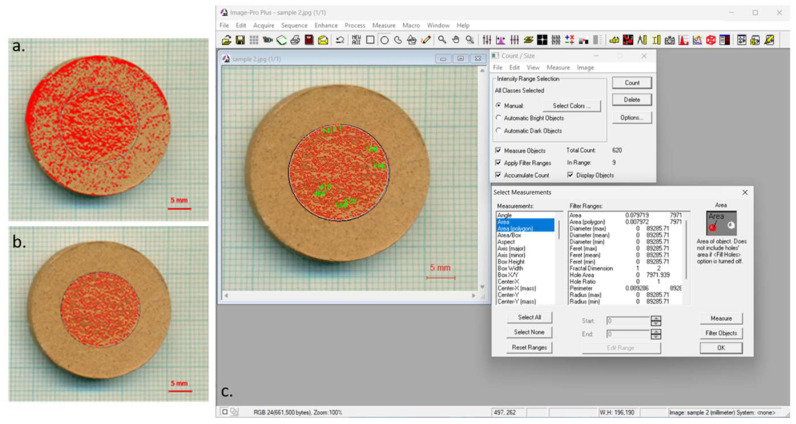
Illustration of the Image-Pro Plus analysis on macrophotograph of the talc-based refractory sample with Igros zeolite exposed to cavitation for 80 min: (**a**) image of the sample, (**b**) image of the sample after using selected measurements, and (**c**) example of selection of parameters on the sample.

The first results of the application of Image-Pro Plus software referred to the determination of the surface degradation level of the samples based on photographs obtained using a scanner with appropriate resolution (1200 dpi). An illustration of the Image-Pro Plus analysis was given on an example macrophotograph (talc-based refractory sample with Igros zeolite after 80 min of cavitation exposure), Figure 9.

**Figure 10:**
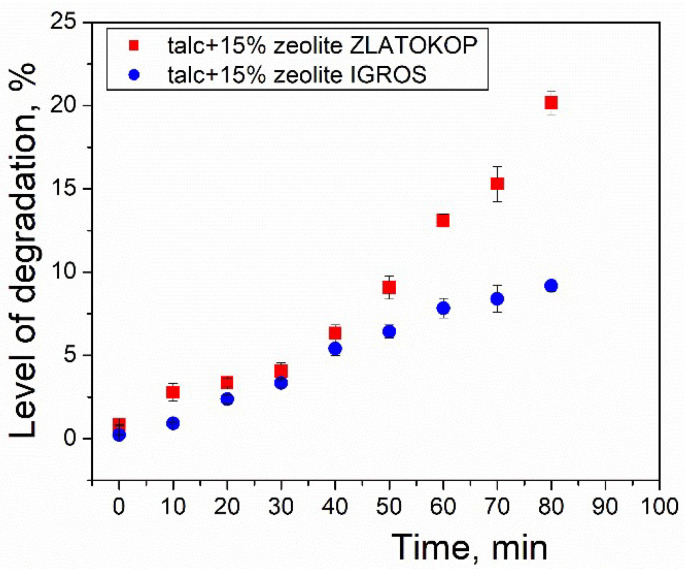
Surface degradation level before and during cavitation exposure of the refractory talc-based samples with Igroš and Zlatokop zeolites.

The level of surface degradation was calculated as the ratio of the damaged area (sum of formed pits’ areas) *P_n_* to the original ideal surface area of the sample (radius 15 mm) *P*_0_. The surface degradation level as a function of cavitation testing time is given in Figure 10.

As seen in Figure 10, during the entire period of cavitation testing, the surface destruction level increases. The samples before cavitation testing do not have detectable damages, which is also evident by visual inspection. After only 10 min of cavitation exposure, surface damage, or the appearance of pits, is observed, and, with time, the number of pits, i.e., the damaged surface area, increases. During the first 40 min of testing, this dependence of both samples is approximately linear, and the values of degradation level can be considered similar.

After 40 min of testing, the refractory samples exhibited different damage progression. While the surface destruction level of talc-based sample with zeolite Igros progresses very slowly, a faster propagation of damage is noticed for talc-based sample with zeolite Zlatokop.

The maximum value of surface destruction level at the end of the testing period of 26.4%, in regard to the total surface area, was recorded for the talc-based sample with zeolite Zlatokop sample. Although its cavitation resistance is lower compared to another sample, these results indicate both refractories’ good resistances to the cavitation’s impact.

It can be concluded that degradation level values agree with mass loss results.

The SEM analysis confirmed the results of mass loss and the level of surface degradation, which indicate the lower resistance to cavitation of the sample with Zlatokop zeolite.

#### 3.3.2. Lifetime Modeling

To introduce a procedure for predicting behavior of talc-based refractory for coating application in cavitating conditions, the idea was to apply the experience with strength degradation modeling based on surface defects of the similar material, alumina-based refractories, with the 0.488 constant and, in the further step, to check the reliability of the obtained model.

Considering the previous attempts at lifetime modeling based on the level of surface degradation [8], a similar model could be given for critical values of mechanical strength of talc-based refractories with zeolite due to cavitation erosion.

The strength degradation can be correlated with the cavitation exposure time or critical dimensions of some of the morphological parameters of formed pits. If a similar approach is used, the strength degradation during time is given by the following equation (Equation (3)):

(3) $$\frac{\sigma }{\sigma_{0}}={\left(\frac{{100-P}_{n}}{P_{0}}\right)}^{0.488}$$

where *σ* is mechanical strength during testing, *σ*_0_ is mechanical strength before testing, *P_n_* is the surface degradation level during the testing (%), and *P*_0_ is the surface degradation level before the testing (%). The constant value of 0.488 has been availed based on the models formulated for alumina-based refractories [8,52].

**Figure 11:**
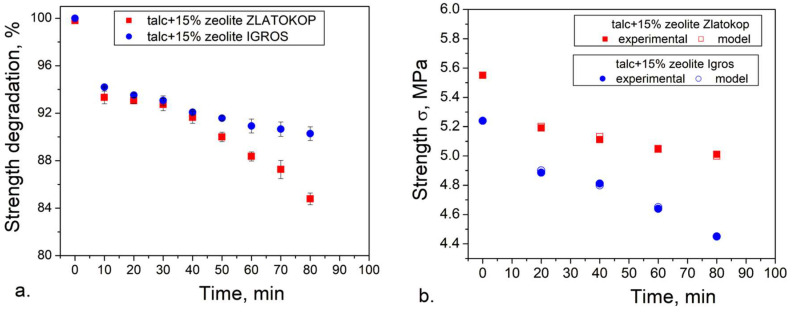
Compressive strength degradation of refractory talc-based samples with zeolites during cavitation testing: (**a**) modeling results, (**b**) validation of the model results by comparing with the experimental values.

The obtained modeling results, with normalized strength values, are given in Figure 11a.

To validate the proposed model, strength was determined experimentally, and the validation results are shown in Figure 11b.

According to the results presented in Figure 11b, good correspondences between model and experimental strength values are evident for both samples. It follows that the proposed strength degradation model is reliable and applicable for the given type of refractory materials.

The critical cavitation conditions can be related to the critical strength value acceptable for the material application. The critical strength value of about 88% of the initial strength has been chosen.

A critical value of 88% of the initial strength is chosen as an example to illustrate how the proposed procedure can be conducted. For various materials in different applications, a higher critical strength can be used, such as 95% of the initial strength value. However, for different chosen strength values, critical time and morphological parameters can be determined as presented. It should be taken into account that special criteria were applied for materials in extreme conditions, which include cavitation. For example, the ASTM standard method for thermal shock resistance determines a critical temperature interval by the mean flexural strength reduction of minimum 30% [53].

**Figure 12:**
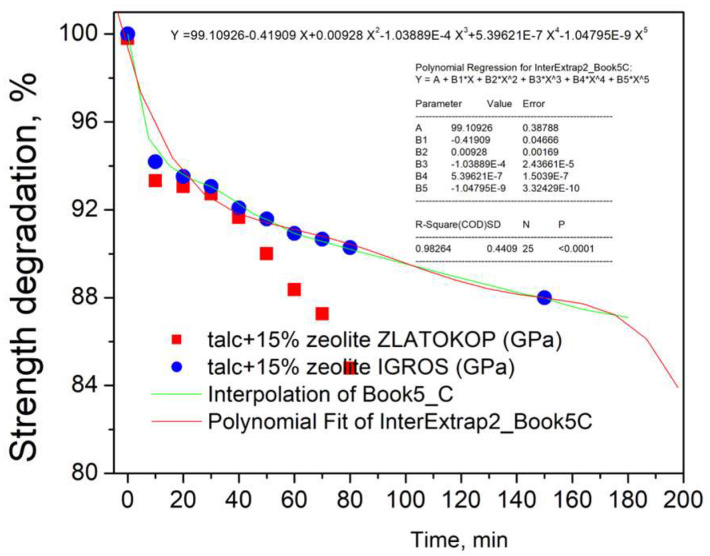
Determination of critical cavitation exposure time for talc-based refractory with Zlatokop zeolite.

In the case of the refractory with zeolite Zlatokop, as seen in Figure 11a, the critical strength of approximately 88% of the initial value corresponds to the cavitation exposure period of 60 min. Unlike for the refractory with zeolite Igros, the critical exposure time when the strength is degraded to 88% of the initial is obtained by extrapolation. Namely, the Origin program was used for extrapolating the level of degradation illustrated in Figure 10. Firstly, the experimental points for degradation level were extrapolated to 25 points in the time range 0–180 min, and, afterward, the polynomial fit was applied. The results are given in Figure 12.

According to the fitting curve in Figure 12, for the chosen critical strength of 88% of the initial, it follows that the corresponding critical exposure time is 150 min.

### 3.4. Monitoring the Interior Damage by Ultrasonic Method

**Figure 13:**
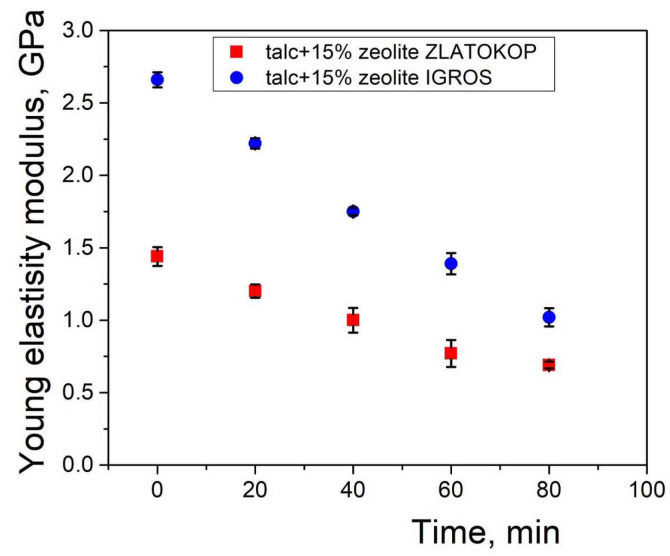
Change in Young’s elasticity modulus before and during cavitation testing of refractory samples composed of 85% talc with 15% zeolite from Igros and Zlatokop deposits.

The obtained values of ultrasonic pulse velocities were used to calculate the Young’s elasticity modulus of both refractories before and during the cavitation exposure. The decline of Young’s elasticity modulus as a function of testing time is presented in Figure 13.

According to the graphs in Figure 13, a decrease in Young’s elasticity modulus during the cavitation exposure is evident. This leads to the conclusion that the damage, in addition to surface progression (Figure 10), also propagates through the interior of the material and increases with the passage of cavitation testing time.

### 3.5. Morphological and Principal Component Analyses

Depending on the expected shape of formed degradation, in this case, pits, different morphological parameters (descriptors) can be used for analysis.

**Figure 14:**
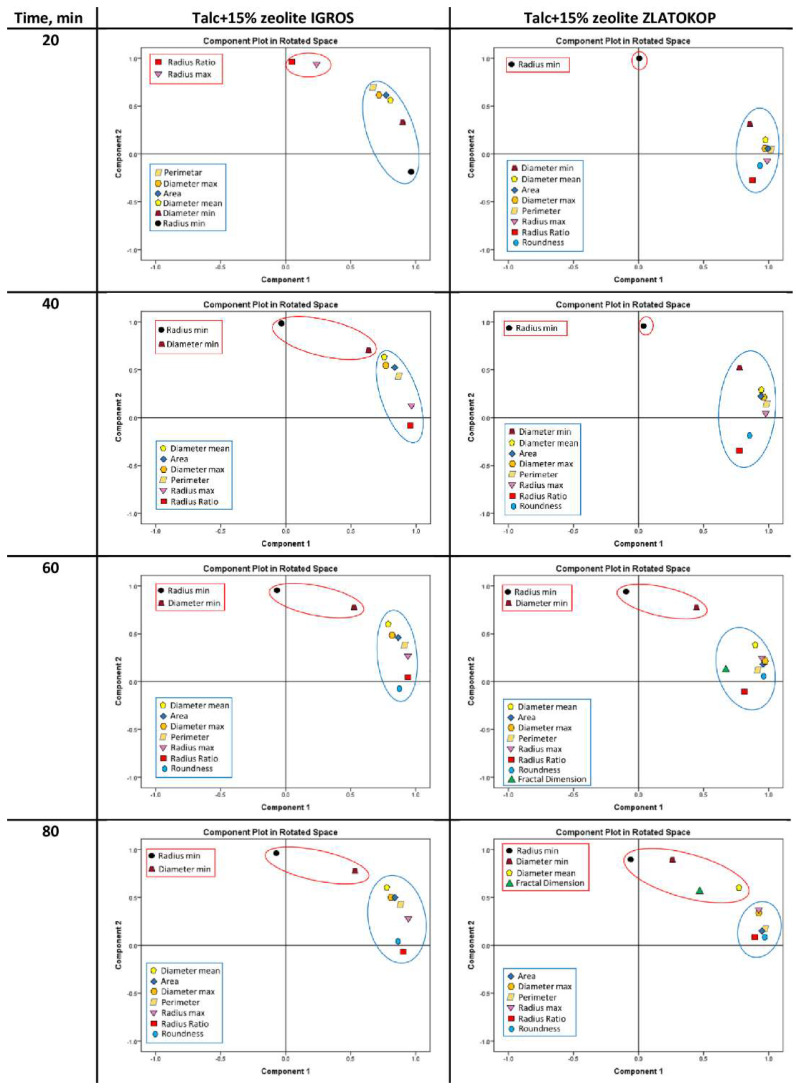
PCA loading plots for talc-based refractory with 15% zeolite.

For the PCA, the following descriptors were used: Area, Diameter (max, mean, and min), Perimeter, Radius (max and min), Radius Ratio, Roundness, and Fractal Dimension (Table 6). PCA was applied to the dataset of values for isolated pits (damages marked in Figure 6) obtained using Image-Pro Plus software. Results for selected morphology parameters and for both tested materials during exposure to cavitation are presented in Figure 14.

Selected descriptors, including Area, Perimeter, Diameter (max, mean, and min), Radius (max and min), Radius ratio, and Roundness proved to be the most informative for both tested materials, so they were subjected to further analysis. The results of the PCA analysis showed that the maximum amount of variance is described by grouping the descriptors into two main components PC1–PC2, whereby most of the parameters are found in PC1, and only a small percentage of the variance is covered by the second component PC2, so those descriptors can be excluded from further analysis.

According to the results of multivariate analysis, the difference with the selected morphological descriptors can be observed by comparing talc-based samples with various zeolite types. For both tested materials and almost the entire testing period, the most informative parameters were Radius (max) and Radius ratio (for the samples with zeolite Igros) or Radius (min) and Diameter (min) (for the samples with zeolite Zlatokop) since these variables had values close to 1 and were strongly correlated with PC2. These results indicated that mentioned parameters should be observed in order to monitor changes in surface defects. The parameters that could be excluded from further analysis were outlined in a red color since these variables had values closer to 0, indicating a weak influence on the PC1. More precisely, they were not sufficiently informative because they did not carry data that could precisely describe the defects.

Regarding the samples with Igros zeolite, at the beginning of the testing, for 10 and 20 min of cavitation exposure, it can be noticed that the damages that arose had very similar shapes since the variances in the selected parameters were almost the same, with variables clustered together and outlined in red. The parameters that could be excluded from further analysis were Radius ratio and Radius (max). After 30 to 80 min of cavitation exposure, Radius (min) could be excluded, as well as the Diameter (min) for 40, 60, and 80 min.

For the samples with Zlatokop zeolite, Radius (min) could be excluded for all exposure periods. With the passage of time, different descriptors could be excluded, for example, for 30, 60, and 70 min Diameter (min). For the period of 80 min, the situation was different, as, in addition to Radius (min), other descriptors (Diameter (mean), and Fractal dimension), could be excluded.

Based on the PCA analysis, it can be concluded that the basic similarity between these two tested materials is that Radius (min) can be excluded for most cavitation exposure periods for both samples, as a parameter that does not contribute significantly to the total variance.

According to the used lifetime model, for the sample with zeolite Igros, the predicted critical exposure time of 150 min indicates its better cavitation resistance compared to the sample containing zeolite Zlatokop, with a critical exposure time of 60 min, based on the strength degradation diagram (Figure 11).

For critical exposure periods, critical morphological parameters can be obtained. Based on the interpolation (for the sample with zeolite Zlatokop) and extrapolation (for the sample with zeolite Igros), some critical morphological descriptors such as Area and Diameter (max and min) were obtained and given in Table 7. (To extrapolate the critical values of the morphological parameters for the sample with zeolite Igros, linear regression in the Origin program was used. After linear fitting, critical values were read for the critical exposure time of 150 min from the fitted curve for each morphological parameter.)

Extrapolation applied for the sample with zeolite Igros only indicated possible values of critical morphological parameters. However, it was very hard to predict these values, considering that two mechanisms—pits formation and pits growth—appeared simultaneously.

According to the presented results, the expected diameters for both refractories had similar values, but the fact is that these dimensions were expected after a much longer time of cavitation when testing for the sample with Igros zeolite. The critical area (average area of the pit) for the sample with Igros zeolite could be much smaller than for the other sample, whereas the differences for the total area were lesser.

**Figure 15:**
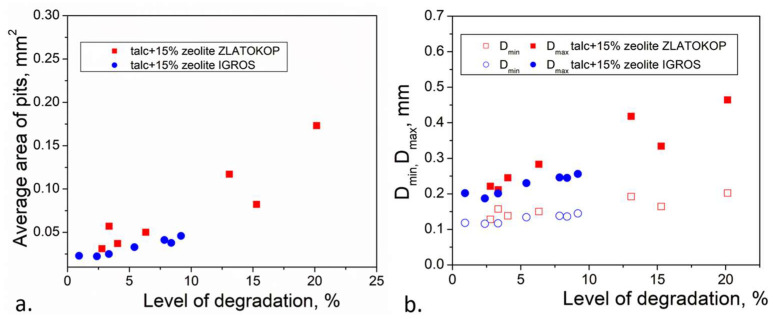
Morphological parameters (**a**) Area (average) and (**b**) D (min) and D (max) against surface degradation level.

As the surface degradation level was used to predict the strength, the variability of the chosen morphological parameter with strength could be presented in diagram Area against the surface degradation level and Diameter against the surface degradation level (Figure 15).

## 4. Conclusions

In this study, behaviors in simulated cavitating conditions of manufactured talc-based refractories with zeolites from two deposits were examined and compared by a proposed methodology that included image and PCA analyses, strength degradation modeling based on surface defects, as well as internal destruction detected by ultrasonic measurements.

The results of mass loss and surface degradation level during the entire cavitation period exhibited trends in continual growth, whereby the values for the refractory sample with Zlatokop zeolite were always higher. The strength degradation model, based on the surface degradation, showed a strength decrease, which was more pronounced for the sample with Zlatokop zeolite. Experimentally determined strength values confirmed the reliability of the model. Principal component analysis indicated morphological parameters that were most informative to describe the defects. For the chosen critical strength, critical cavitation exposure period was determined along with critical morphological descriptors such as Area and Diameter (max and min) afterwards. The declines in ultrasonic pulse velocities, and Young’s elasticity modulus accordingly, indicated that the damage impacts spread from the surfaces towards the material’s interior. According to the XRD analysis of the sintered refractories, higher resistance to the cavitation erosion of the sample with Igros zeolite can be ascribed to the presence of the small amount of the Mg_2_Al_2_O_5_ phase, which was not detected in the sample with Zlatokop zeolite.

By compiling different approaches, consistent results were obtained, and the possibility of using talc-based refractories with zeolite as protective coatings under cavitation conditions was assessed. The proposed procedure and methodology for estimating refractory’s resistance in simulated cavitating conditions can be useful for industrial applications and predictions of sample behaviors in industrial control laboratories, but it should be adjusted and verified in real conditions. Also, this methodology could be applied for different materials and other processes that cause surface damage.

## Figures and Tables

**Table 1:** Chemical composition of talc.

Component	Content (%)
SiO_2_	59.70
MgO	28.93
Al_2_O_3_	4.64
CaO	1.12
Fe_2_O_3_	1.18
MnO	0.085
Na_2_O	0.016
K_2_O	0.012
Ba	0.01
Zn	0.0035
Ni	0.0027
L.O.I.	4.21

**Table 2:** Properties of talc.

Parameter	Value
Bulk density, kg/m^3^	2.7–2.8
Melting point, °C	1450–1550
Maximum service temperature, °C	1350
Refractoriness, SK/1450 °C	14
Linear expansion coefficient at 25 °C, cm/cm °C	2.7 × 10^−6^
Thermal conductivity coefficient at 27 °C, W/cm K	0.035–0.040

**Table 3:** Average chemical composition of zeolite from the Igros deposit.

Component	Content (%)
SiO_2_	63.56
Al_2_O_3_	15.35
CaO	4.67
Fe_2_O_3_	2.00
MgO	1.49
Na_2_O	1.16
K_2_O	0.92
TiO_2_	0.357
MnO	0.016
Pb	0.0046
Cu	0.011
Zn	0.0053
Sb	0.004
Cr	0.0003
Cd	0.0003
L.O.I.	10.42

**Table 4:** Average chemical composition of zeolite from the Zlatokop deposit.

Component	Content (%)
SiO_2_	65.79
Al_2_O_3_	10.91
CaO	3.89
Fe_2_O_3_	1.96
MgO	1.27
K_2_O	0.97
Na_2_O	0.82
TiO_2_	0.39
MnO	0.18
Cu	0.008
Zn	0.008
Sb	0.0043
Cr	0.0002
Cd	0.0002
L.O.I	13.42

**Table 5:** XRD quantitative analysis of talc-based refractories with zeolites.

	Sample with Igros Zeolite	Sample with Zlatokop Zeolite
MgSiO_3_	93.5%	95%
SiO_2_	2.6%	5%
Mg_2_Al_2_O_5_	3.9%	-

**Table 6:** Selected descriptors for the morphological characterization of formed pits.

Morphological Descriptor	Definition	Image
Area	Area of object. Does not include holes area.	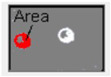
Diameter (max)	Length of the longest line joining two points of objects outline and passing through the centroid.	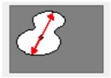
Diameter (mean)	Average length of diameters measured at 2-degree intervals and passing through objects centroid.	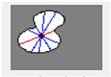
Diameter (min)	Length of the shortest line joining two points of objects outline and passing through the centroid.	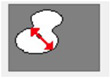
Perimeter	Length of the object outline.	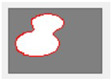
Radius (max)	Maximum distance between objects centroid and outline.	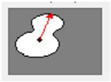
Radius (min)	Minimum distance between objects centroid and outline.	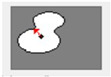
Radius Ratio	Ratio between Radius (max) and Radius (min).	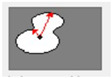
Roundness	Ratio of the surface area of an object to the area of the circle whose diameter is equal to the maximal diameter of the object.	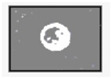
Fractal Dimension	Represents the fractal dimension of the object contour.	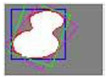

**Table 7:** Critical values of morphological parameters.

	Sample with Zlatokop Zeolite	Sample with Igros Zeolite
Critical time, min	60	150
Area (average), mm^2^	0.117	0.068
Area (total), mm^2^	26.33	36.43
Diameter (max), mm	0.418	0.330
Diameter (min), mm	0.192	0.175

## Data Availability

The data presented in this study are available on request from the corresponding author.
